# Complete genome sequence of *Syntrophobacter fumaroxidans* strain (MPOB^T^)

**DOI:** 10.4056/sigs.2996379

**Published:** 2012-09-26

**Authors:** Caroline M. Plugge, Anne M. Henstra, Petra Worm, Daan C. Swarts, Astrid H. Paulitsch-Fuchs, Johannes C.M. Scholten, Athanasios Lykidis, Alla L. Lapidus, Eugene Goltsman, Edwin Kim, Erin McDonald, Lars Rohlin, Bryan R. Crable, Robert P. Gunsalus, Alfons J.M. Stams, Michael J. McInerney

**Affiliations:** 1 Laboratory of Microbiology, Wageningen University, Wageningen, Netherlands; 2 Department of Microbiology, Immunology, and Molecular Genetics, University of California, Los Angeles, CA, USA; 3 Wetsus, Centre of Excellence for Sustainable Water Technology, Leeuwarden, Netherlands; 4 Microbiology Group, Pacific Northwest National Laboratory, Richland, WA, USA; 5 Joint Genome Institute, Walnut Creek, CA, USA; 6 Department of Botany and Microbiology, University of Oklahoma, Norman, OK, USA

**Keywords:** Anaerobic, Gram-negative, syntrophy, sulfate reducer, mesophile, propionate conversion, host-defense systems, *Syntrophobacteraceae*, *Syntrophobacter fumaroxidans*, *Methanospirillum hungatei*

## Abstract

*Syntrophobacter fumaroxidans* strain MPOB^T^ is the best-studied species of the genus *Syntrophobacter*. The species is of interest because of its anaerobic syntrophic lifestyle, its involvement in the conversion of propionate to acetate, H_2_ and CO_2_ during the overall degradation of organic matter, and its release of products that serve as substrates for other microorganisms. The strain is able to ferment fumarate in pure culture to CO_2_ and succinate, and is also able to grow as a sulfate reducer with propionate as an electron donor. This is the first complete genome sequence of a member of the genus *Syntrophobacter* and a member genus in the family *Syntrophobacteraceae*. Here we describe the features of this organism, together with the complete genome sequence and annotation. The 4,990,251 bp long genome with its 4,098 protein-coding and 81 RNA genes is a part of the Microbial Genome Program (MGP) and the Genomes to Life (GTL) Program project.

## Introduction

Strain MPOB^T^ (DSM 10017) is the type strain of *Syntrophobacter fumaroxidans* [1], which is one of the four described species within the genus of *Syntrophobacter* [2]. The type species of the genus *Syntrophobacter* is *Syntrophobacter wolinii* (DSM 2805) [2,3]. Strain MPOB^T^ is currently the best-studied species in the genus *Syntrophobacter*. The genus name derives from the Greek words “*syn*”, together with “*troph*”, one who feeds, and “bacter”, rod shaped, referring to a rod-shaped bacterium growing in syntrophic association with hydrogen- and formate-scavenging microorganisms [1]. The species epithet derives from the Latin word “fumaricum” pertaining to fumaric acid and the Latin adjective “oxidans”, oxidizing, referring to fumarate fermentation.

Strain MPOB^T^ was isolated from granular sludge of a mesophilic upflow anaerobic sludge blanket (UASB) reactor, treating waste from a sugar refinery [1].

All currently identified syntrophic propionate-oxidizing bacteria are affiliated either with the class *Deltaproteobacteria* within the phylum *Proteobacteria* [4], to which *Syntrophobacter* belongs, or the class *Clostridia* within the phylum *Firmicutes* [5-7]. Many of the *Syntrophobacter* spp. are able to use sulfate as the electron acceptor for propionate oxidation and some other organic compounds and hydrogen [4,8]. In addition, they can grow by fermentation of pyruvate and fumarate. *Smithella propionica* is phylogenetically related to the genus *Syntrophus* [9] but lacks the ability to reduce sulfate. It also uses a different pathway to oxidize propionate distinct from that used by *Syntrophobacter* strains, which one that possibly involves a six-carbon intermediate. It can also grow on crotonate in pure culture [9,10].

Here we describe the features of *Syntrophobacter fumaroxidans* strain MPOB^T^ together with the complete genome sequence and annotation.

## Classification and features

**Figure 1:**
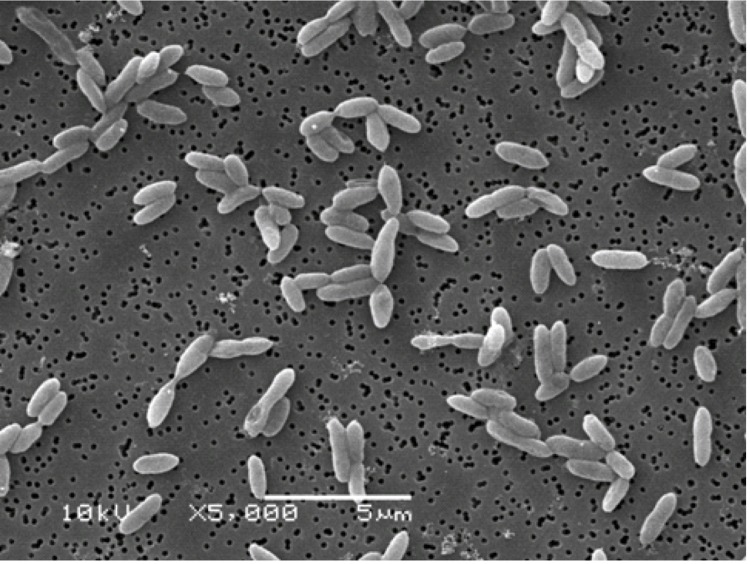
Scanning electron micrograph of *S. fumaroxidans* during exponential phase of growth.

Cells of *S. fumaroxidans* strain MPOB^T^ are short rods with rounded ends of 1.1-1.6 ×1.8-2.5 µm (Figure 1 and Table 1). Cells are Gram-negative, non-motile, and do not form endospores. The metabolism is strictly anaerobic and can be respiratory or fermentative [2,4]. The temperature range for growth is 20-40°C (optimum at 37°C).

**Table 1:** Classification and general features of *S. fumaroxidans* MPOB^T^ according to the MIGS recommendations [11].

**MIGS ID**	**Property**	**Term**	**Evidence code**
	Current classification	Domain *Bacteria*	TAS [12]
		Phylum *Proteobacteria*	TAS [13]
		Class *Deltaproteobacteria*	TAS [14,15]
		Order *Syntrophobacterales*	TAS [14,16]
		Family *Syntrophobacteraceae*	TAS [14,17]
		Genus *Syntrophobacter*	TAS [3,18,19]
		Species *Syntrophobacter fumaroxidans*	TAS [1]
		Type strain MPOB	TAS [1,2]
	Gram stain	Negative	TAS [1]
	Cell shape	rod-shaped	TAS [1]
	Motility	non-motile	TAS [1]
	Sporulation	non-sporulating	TAS [1]
	Temperature range	Mesophilic, 20-40^0^C	TAS [1]
	Optimum temperature	37°C	TAS [1]
MIGS-22	Oxygen requirement	strictly anaerobic	TAS [1]
	Carbon source	In pure culture: fumarate, malate, aspartate and pyruvate, fumarate + propionate, H_2_ + fumarate, formate + fumarate fumarate + sulfate, H_2_ + sulfate, formate + sulfate In syntrophy with hydrogen and formate scavenger, propionate	TAS [1]
	Energy source	Propionate, fumarate, malate, aspartate, pyruvate, hydrogen, formate	TAS [1]
MIGS-6	Habitat	Fresh water sediments, Anaerobic bioreactors	[1,4]
MIGS-15	Biotic relationship	Free-living	NAS
MIGS-14	Pathogenicity	Not reported	NAS
	Biosafety level	Not reported	NAS
	Isolation	Granular sludge from a mesophilic upflow anaerobic sludge blanket UASB) reactor treating sugar refinery waste	TAS [1]
MIGS-4	Geographic location	Breda, the Netherlands	TAS [1]
MIGS-5	Sample collection time	1987	IDA
MIGS-4.1	Latitude	51^°^35′42.55′′ N	IDA
MIGS-4.2	Longitude	4^°^46′12.11′′ E	IDA
MIGS-4.3	Depth	not reported	NAS
MIGS-4.4	Altitude	not reported	NAS

Evidence codes – IDA: Inferred from Direct Assay (first time in publication); TAS: Traceable Author Statement (i.e., a direct report exists in the literature); NAS: Non-traceable Author Statement (i.e., not directly observed for the living, isolated sample, but based on a generally accepted property for the species, or anecdotal evidence). These evidence codes are from the Gene Ontology project [20]. If the evidence code is IDA, then the property was directly observed for a live isolate by one of the authors or an expert mentioned in the acknowledgements.

Strain MPOB^T^ utilizes propionate syntrophically via the methylmalonyl-CoA pathway in co-culture with the hydrogen and formate-utilizing methanogen, *M. hungatei*, and in pure culture using sulfate or fumarate as an electron acceptor [21,22]. In these cases, propionate is converted stoichiometrically to acetate and CO_2_ with concomitant production of methane, sulfide or succinate, respectively [1,22]. Thiosulfate also serves as an electron acceptor, but nitrate is not utilized. Strain MPOB^T^ ferments fumarate to succinate and CO_2_ using the acetyl-CoA cleavage pathway [22], and reduces fumarate to succinate with hydrogen or formate as the electron donor [21,23].

**Figure 2:**
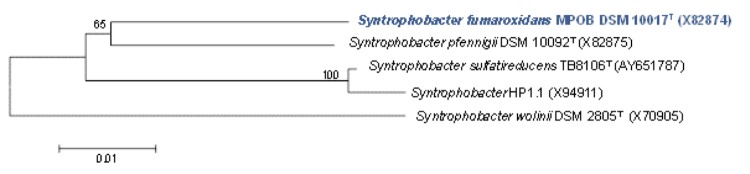
Neighbor-joining tree, based on 16S rRNA gene sequences, highlighting the position of *Syntrophobacter fumaroxidans* strain MPOB DSM 10017^T^ relative to other *Syntrophobacter* species. Numbers above branches are support values from 1,000 bootstrap replicates if larger than 60%. Strain MPOB, with a genome-sequencing project registered in GOLD [24] printed in blue. Bar indicates 0.01 substitutions per nucleotide position.

Figure 2 shows the phylogenetic neighborhood of *S. fumaroxidans* strain MPOB^T^ in a 16S rRNA gene-based tree. This tree shows that these Gram-negative syntrophic propionate oxidizers form one cluster within the family of *Syntrophobacteraceae* of the order *Syntrophobacterales*. All propionate-degrading bacteria from the order *Syntrophobacterales* are capable of propionate degradation in syntrophic coculture with a syntrophic partner but also as a pure culture coupled to dissimilatory sulfate reduction [8]. The physiological and genomic data on microorganisms capable of syntrophy from the *Syntrophobacterales*, which are abundantly present in methanogenic environments [25-28], indicate that all have retained their sulfate-reducing capability [22,29,30]. *Syntrophobacterales* contains sulfate-reducing species that are capable of syntrophy, and growth by sulfate reduction, as well as species capable of syntrophy that contain the bisulfite reductase genes (*dsrAB*), but are not capable of reducing sulfate; indicating an evolutionary connection between the sulfate-reducing and syntrophic lifestyles [8].

The syntrophic species in the *Syntrophobacterales* can be divided in two groups based on their ability to reduce sulfate or not, which suggests an evolutionary connection between the sulfate-reducing and syntrophic lifestyles [8].

Two 16S rRNA gene sequences are present in the genome of strain MPOB^T^. Sequence analysis indicates that these two genes are almost identical (2 bp difference), and that both genes differ by up to 8 nucleotides from the previously published 16S rRNA gene sequence (X82874).

### Chemotaxonomy

*S. fumaroxidans* strain MPOB^T^ contains *c*- and *b*- type of cytochromes and the menaquinones MK-6 and MK-7 [1].

## Genome sequencing and annotation

### Genome project history

This organism was selected for sequencing on the basis of its syntrophic and sulfate-reducing lifestyles and its phylogenetic position, and is part of the Microbial Genome Program (MGP) and the Genomes to Life (GTL) Program. The genome project is deposited in the Genomes OnLine Database [Gc00453] and the complete genome sequence (CP000478) is deposited in GenBank. Sequencing, finishing and annotation were performed by the DOE Joint Genome Institute (JGI). A summary of the project information is shown in Table 2.

**Table 2:** Genome sequencing project information

**MIGS ID**	**Property**	**Term**
MIGS-31	Finishing quality	Finished
MIGS-28	Libraries used	3kb (pUC18c), 8kb (pMCL200) and 40kb (pcc1Fos)
MIGS-29	Sequencing platforms	Sanger
MIGS-31.2	Sequencing coverage	7×
MIGS-20	Assemblers	PGA
MIGS-32	Gene calling method	Prodigal, GenePRIMP
	INSDC / Genbank ID	CP000478
	Genbank Date of Release	October 27, 2006
	GOLD ID	Gc00453
	NCBI project ID	13013
	Database: IMG	[31]
MIGS -13	Source material identifier	DSM 10017
	Project relevance	Genomes to Life: Bioreactors, Biotechnology, Carbon cycle, Energy production, Hydrogen production

### Growth conditions and DNA isolation

*S. fumaroxidans* MPOB^T^ was grown at 37^o^C in anaerobic bicarbonate buffered mineral salts medium as was described previously [23]. High molecular weight genomic DNA was isolated from 2 -2.5 g concentrated cell pellets using the CTAB method recommended by Joint Genome Institute (JGI), which can be found at the JGI website [32].

### Genome sequencing and assembly

*Syntrophobacter fumaroxidans* genomic DNA was sequenced at JGI using a combination of 3 kb, 8 kb and 40 kb DNA libraries. All general aspects of library construction and sequencing performed at the JGI can be found at the JGI website [32]. The Phred/Phrap/Consed software package [33] was used to assemble all three libraries and to assess quality [34-36]. Possible misassemblies were corrected, and gaps between contigs were closed by editing in Consed, custom primer walks or PCR amplification (Roche Applied Science, Indianapolis, IN). The error rate of the completed genome sequence of *S. fumaroxidans* is less than 1 in 50,000. Pair-wise graphical alignments of whole genome assemblies (e.g. synteny plots) were generated by using the MUMmer system [37,38].

### Genome annotation

Automated gene prediction was performed by using the output of Critica [39] complemented with the output of the Generation and Glimmer models [37]. The predicted CDSs were translated and used to search the National Center for Biotechnology Information (NCBI) nonredundant database, UniProt, TIGRFam, Pfam, PRIAM, KEGG, COG, and InterPro databases. Additional gene prediction analysis and functional annotation was performed within the Integrated Microbial Genomes-Expert Review platform [40].

## Genome properties

**Figure 3:**
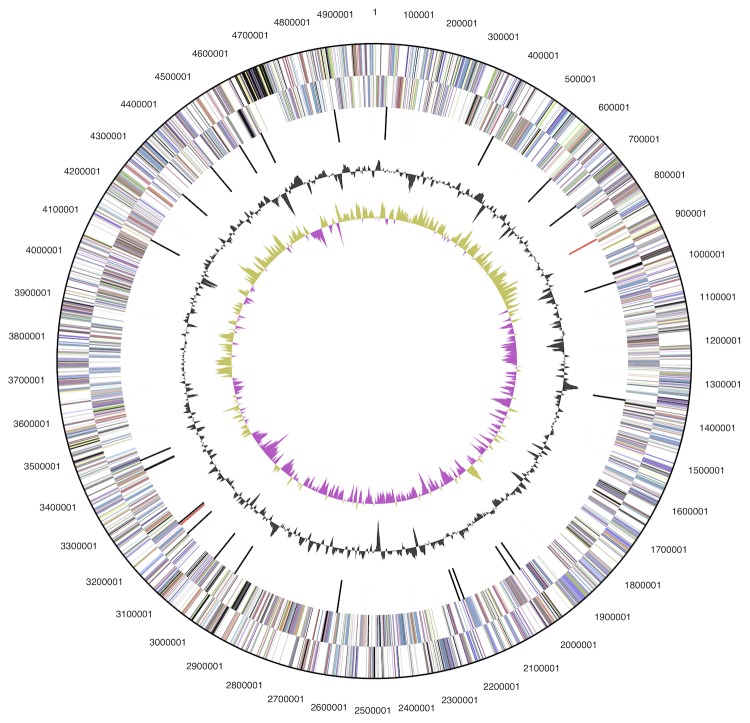
Graphical circular map of the genome. From outside to the center: Genes on for-ward strand (color by COG categories), Genes on reverse strand (color by COG categories), RNA genes (tRNAs green, rRNAs red, other RNAs black), GC content, GC skew.

The genome is 4,990,251 bp long and contains one circular chromosome with a 59.95% GC content (Figure 3). Of the 4,179 genes predicted, 4,098 were protein coding genes, 81 RNAs and 34 pseudogenes. A total of 67.2% of the genes were assigned a putative function while the remaining ones were annotated as hypothetical proteins. The properties and the statistics of the genome are summarized in Table 3. The distribution of genes into COGs functional categories is presented in Table 4.

**Table 3:** Genome Statistics

**Attribute**	**Value**	**% of Total**
Genome size (bp)	4,990,251	100
DNA coding region (bp)	4,115,129	82.46
DNA G+C content (bp)	2,991,592	59.95
Number of replicons	1	
Extrachromosomal elements	0	
Total genes	4,179	100
RNA genes	81	1.94
rRNA operons	6	0.14
Protein-coding genes	4,098	98.06
Pseudo genes	34	0.81
Genes with function prediction	1,289	30.85
Genes in paralog clusters	791	18.93
Genes assigned to COGs	2,959	70.81
Genes assigned Pfam domains	3,075	73.58
Genes with signal peptides	741	17.73
Genes with transmembrane helices	1,035	24.77
CRISPR repeats	4	

**Table 4:** Number of genes associated with the general COG functional categories

**Code**	**value**	**% age**	**Description**
J	170	5.18	Translation, ribosomal structure and biogenesis
A	1	0.03	RNA processing and modification
K	153	4.66	Transcription
L	139	4.24	Replication, recombination and repair
B	4	0.12	Chromatin structure and dynamics
D	32	0.98	Cell cycle control, cell division, chromosome partitioning
Y	0	0.00	Nuclear structure
V	54	1.65	Defense mechanisms
T	263	8.02	Signal transduction mechanisms
M	229	6.98	Cell wall/membrane/envelope biogenesis
N	35	1.07	Cell motility
Z	0	0.00	Cytoskeleton
W	0	0.00	Extracellular structures
U	89	2.71	Intracellular trafficking, secretion, and vesicular transport
O	145	4.42	Posttranslational modification, protein turnover, chaperones
C	375	11.43	Energy production and conversion
G	137	4.18	Carbohydrate transport and metabolism
E	274	8.35	Amino acid transport and metabolism
F	67	2.04	Nucleotide transport and metabolism
H	178	5.43	Coenzyme transport and metabolism
I	95	2.90	Lipid transport and metabolism
P	156	4.75	Inorganic ion transport and metabolism
Q	47	1.43	Secondary metabolites biosynthesis, transport and catabolism
R	407	12.40	General function prediction only
S	231	7.04	Function unknown
-	1220	29.19	Not in COGs

## Comparison to other genomes

**Figure 4:**
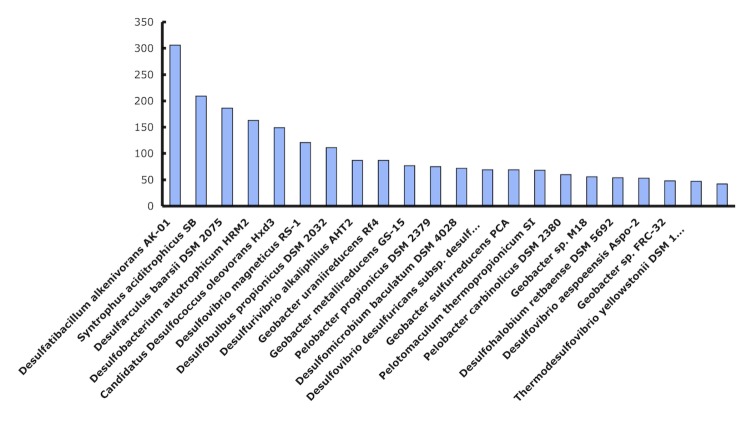
Best reciprocal protein hits for *S. fumaroxidans* ORFs with other genomes.

The *S. fumaroxidans* genome is intermediate in size for the *Deltaproteobacteria* genomes sequenced thus far, which range in size from 1.72 to 13.03 Mbp. Notably, it has 1.8 Mbp more DNA than the other well studied syntrophic fatty acid degrader, *Syntrophus aciditrophicus* SB (3.1 Mbp). When *S. fumaroxidans* ORFs were compared on a pair-wise basis to individual microbial genomes, the best reciprocal BLAST hits revealed the closest associations to the following *Deltaproteobacteria*: *Desulfobacterium autotrophicum* HRM2 (1593 reciprocal gene hits), *Desulfatibacillum alkenivorans* AK-01 (1551), and *Desulfovibrio magneticus* RS-1 (1448) (Figure 4). Approximately 1,200 genes are similar, and are well conserved across the 25 Gram-negative species shown. The remaining genes (ca. 2,400) represent a novel complement within the *S. fumaroxidans* genome. Notably, *Pelobacter propionicus* SSM 2379 was the 18^th^ closest to *S. fumaroxidans*. Although *P. propionicus* is not known to grow syntrophically, other *Pelobacter* species oxidize various alcohols syntrophically [4].

**Figure 5:**
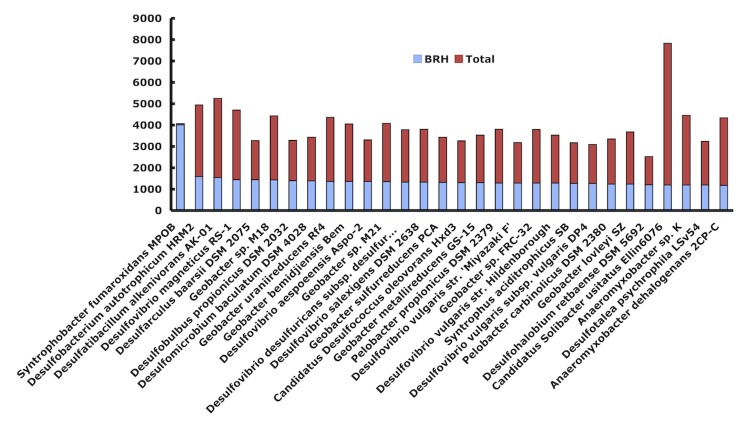
Best Blast hit distribution of *S. fumaroxidans* ORFs with other genomes. In blue the best reciprocal hits; in red the total coding DNA sequences (CDS)Insights into the genome

In another comparison, the best BLAST hit to any microbial gene was determined (Figure 5) and showed only 306, 209, and 186 closest hits to the genomes of *D. alkenivorans* AK-01, *S. aciditrophicus* SB, and *Desulfarculus baarsii* DSM 2075, respectively. Interestingly, no archaeal genomes were identified that suggest the possibility of lateral gene transfer events from these potential syntrophic partners. However, a recent study using an experimental approach and a different bioinformatic platform, the STRING database, which includes known and predicted protein–protein interactions [41,42], discovered that a gene set present in syntrophic bacteria such as *Pelobacter carbinolicus*, *S. fumaroxidans*, *Syntrophomonas wolfei*, and *Syntrophus aciditrophicus*, tended to be clustered with their homologues in archaeal genera, and they were rooted on archaeal species in the constructed phylogenetic trees. This suggests that they were horizontally transferred from archaeal methanogens [43]. Gene products for this gene set are hypothetical.

### Carbon flow and electron transfer

**Figure 6:**
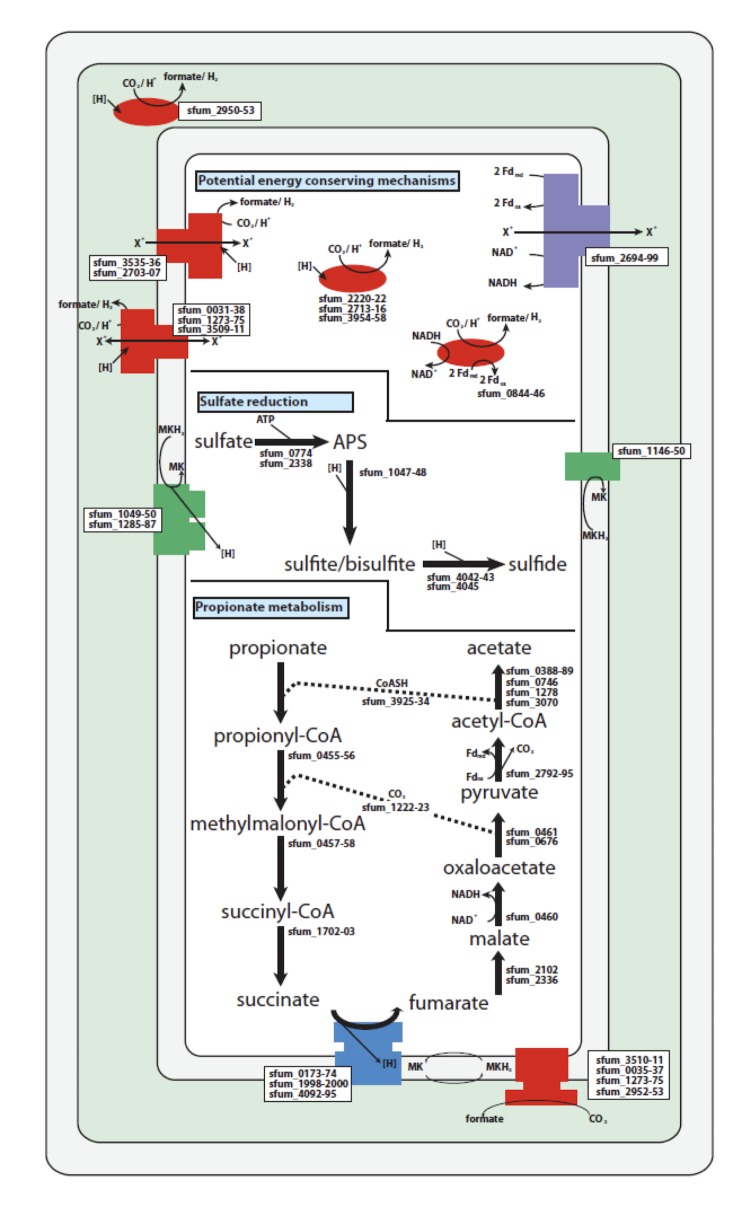
Metabolic reconstruction of propionate metabolism of *S. fumaroxidans*.

The genome of *S. fumaroxidans* contains genes predicted to encode enzymes that are necessary for the conversion of propionate to acetate and carbon dioxide by the methylmalonyl-CoA pathway (Figure 6). Propionyl-CoA:acetate HSCoA transferase (Sfum_3933) is present as part of a gene cluster encoding several presumably redox active proteins. Two genes annotated as electron transfer flavoprotein (ETF) alpha subunits (Sfum_3928-29) and a gene annotated as an ETF beta subunit (Sfum_3930) are part of this gene cluster, as are genes for a putative hydroxylase (Sfum_3932) and an acyl-CoA dehydrogenase (Sfum_3931). Additionally, a gene annotated as an FAD-dependent oxidoreductase (Sfum_3927) and one encoding a 4Fe-4S ferredoxin-binding domain (Sfum_3926) are part of this gene cluster. Genes predicted to encode a sigma 54-dependent transcriptional regulator (Sfum_3934) and a protein belonging to the major facilitator superfamily (MFS) flank this gene cluster upstream and downstream, respectively.

Propionyl-CoA is converted first to (S)-methylmalonyl-CoA and then to (R)-methylmalonyl-CoA by methylmalonyl-CoA epimerase (Sfum_0455), which is part of a two-gene cluster and pairs with a gene predicted to code for an ATP-dependent amino acid transport protein (Sfum_0456). This gene cluster appears immediately downstream of a two-gene cluster predicted to encode methylmalonyl-CoA mutase (Sfum_0457-8). Sfum_1223 may encode a carboxyltransferase involved in cycling carbon dioxide between the decarboxylation of oxaloacetate and the carboxylation of propionyl-CoA to form methylmalonyl-CoA. Methylmalonyl-CoA mutase (Sfum_0457-8) then converts methylmalonyl-CoA to succinyl-CoA. Removal of the CoA group is presumably accomplished by a succinyl-CoA synthetase (Sfum_1702-03), which forms succinate coupled to ATP formation from ADP and phosphate.

Succinate is then further oxidized to fumarate by a membrane-bound succinate dehydrogenase/fumarate reductase. Succinate oxidation via a menaquinone to fumarate is the most energy dependent reaction in the methyl-malonyl-CoA pathway propionate degradation to acetate in *S. fumaroxidans.* Succinate oxidation via menaquinone is endergonic since the midpoint potential of succinate is more positive (+30 mV) than the menaquinone (-80 mV). Therefore, the reaction requires a transmembrane proton gradient to function. Schink [44] calculated that about 0.66 ATP has to be invested to make this reaction energetically possible at a hydrogen partial pressure of1Pa and formate concentrations of 10 μM, which can be maintained by methanogens in a syntrophic community. The genome of *S. fumaroxidans* contains four gene clusters *sdh*ABC (Sfum_1998-2000), *frd*ABEF (Sfum_4092-4095), *sdh*AB-1 (Sfum_0172-0174) and *sdh*AB-2 (Sfum_2103-2104) with sequence similarity to succinate dehydrogenases or fumarate reductase. *S. fumaroxidans* might use separate enzymes for succinate oxidation and fumarate reduction. During growth with propionate plus fumarate, *S. fumaroxidans* needs an active fumarate reductase, whereas during growth with propionate plus sulfate, or during syntrophic growth on propionate an active succinate dehydrogenase is required [21].

In *S. fumaroxidans*, the gene cluster *sdhABC* (Sfum_1998-2000) codes for two cytoplasmic subunits (*sdhA* and *B*) and a five-trans-membrane (5TM) subunit containing heme (*sdhC*) which is similar to the type B trans-membrane subunit of *Wolinella succinogenes* Frd and *Bacillus subtilis* Sdh (Hägerhäll 1997). SdhA contains the conserved catalytic core residues and SdhB contains motifs for binding of three iron sulfur clusters, [2Fe2S], [4Fe4S], and [3Fe4S].

The gene cluster *frdABEF* (Sfum_4092 - 4095) of *S. fumaroxidans* lacks a gene coding for the trans-membrane subunit and is therefore classified as a type E Frd [45]. The type E succinate:quinone oxidoreductases differ from the other four types in that they do not contain heme and have two hydrophobic subunits, SdhE and SdhF. Rather, the type E succinate:quinone oxidoreductases are more similar to those in cyanobacteria (*Synechocystis*) and the heterodisulfide reductases from methanogens such as *Methanocaldococcus jannaschii* (formerly known as *Methanococcus jannaschii*) [46]. Like other gene clusters coding for E-type Frds, *frdABEF* contains *frdE* that codes for a cysteine-rich domain involved in cytochrome binding and membrane attachment [46]. The function of the protein encoded by *frdF* is unknown [46]. FrdA contains the conserved catalytic residues; FrdB contains motifs for binding of two iron sulfur clusters, [2Fe2S] and [4Fe4S]. The lack of [3Fe4S] may be compensated by iron sulfur clusters present in FrdE and F.

The third gene cluster with similarity to succinate:quinone oxidoreductases (*sdhAB-1*) lacks a gene coding for a transmembrane domain. SdhA contains the conserved catalytic residues and the SdhB contains a motif for [2Fe2S] iron sulfur cluster binding, but lacks motifs for [4Fe4S] or [3Fe4S] binding. Therefore, the required electron transfer via three iron-sulfur clusters cannot occur, meaning that *sdh*AB does not code for a conventional Sdh or Frd [45]. Transmembrane proton transfer may occur through cytochromes for which a variety of genes are present in the genome: *cytcb_561_* (Sfum_0090-91); *cytc_3_* (Sfum_4047); *cydAB-1* (Sfum_3008-09); *cydAB-2* (Sfum_0338-39); *cytb5* (Sfum_3227); *cytb* (Sfum_2932).

The fourth gene cluster is *sdh*AB-2 (Sfum_2103-04). These genes are part of a larger gene cluster that contains genes predicted for aconitase (Sfum_2106), citrate synthase (Sfum_2105), fumarase (Sfum_2102) and a ubiquinone prenyl transferase (Sfum_2101). These enzymes most likely function to supply biosynthetic intermediates. A 348 bp hypothetical protein (Sfum_2100) was also detected as part of this gene cluster.

The next step is the hydroxylation of fumarate forming malate (Figure. 6). Two genes for fumarase were detected in the genome. As discussed previously, a fumarase-encoding gene (Sfum_2102) is a part of a gene cluster containing aconitase, citrate synthase, succinate dehydrogenase and ubiquinone prenyl transferase encoding genes. A second fumarase-encoding gene (Sfum_2336) does not appear to be part of a gene cluster.

Malate is oxidized to oxaloacetate by malate dehydrogenase (Sfum_0460) and oxaloacetate is decarboxylated to pyruvate by pyruvate carboxyltransferase (Sfum_0461, 0676). The decarboxylation of oxaloacetate to pyruvate is concomitant with a carboxyl transfer reaction to form methylmalonyl-CoA from propionyl-CoA (Sfum_1223). Acetyl-CoA is formed from pyruvate by pyruvate ferredoxin oxidoreductase (Sfum_2792-95) and the CoA moiety is recycled to activate propionate to propionyl-CoA. Acetyl-CoA could also be converted to acetyl-phosphate and acetate by phosphotransacetylase and acetate kinase encoded by Sfum_1472-73. Formation of acetate from acetyl-phosphate could result in ATP synthesis by substrate-level phosphorylation. However, when strain MPOB^T^ was grown on propionate, the activity of this enzyme was below detection level, suggesting that acetate was formed exclusively via an acetyl-CoA: propionate HS-CoA transferase [22].

Taken together, the genome reveals a complete set of genes for the conversion of propionate to acetate and carbon dioxide by the methylmalonyl-CoA pathway. Also present are genes and gene clusters for electron transfer from the key carbon oxidation steps leading to hydrogen and formate formation when *S. fumaroxidans* grows syntrophically (Figure. 6). These include the previously discussed fumarate reductase for quinone reduction from succinate. Menaquinol would then, ostensibly, shuttle electrons to a membrane-bound formate dehydrogenase (carbon dioxide reductase) (Sfum_0030-1) or hydrogenase complexes (Sfum_2220-22 and Sfum_2713-16). No formate dehydrogenase genes with transmembrane helices were predicted. However, several genes coding for cytochromes were detected in the genome, which do not appear to be part of larger gene clusters. These cytochromes may provide a platform for formate dehydrogenase subunits to receive electrons from the menaquinol pool. In addition, the cytochromes may also play a role in sulfate reduction, similar to *Desulfovibrio* sp [47]. Reducing equivalents generated by cytosolic events, such as the oxidation of malate to oxaloacetate and pyruvate to acetyl-CoA and CO_2_, are probably NAD(P)H and reduced ferredoxin, respectively. Several soluble cytosolic confurcating hydrogenases (Sfum_0844-46) and formate dehydrogenases (Sfum_2703-07) probably catalyze hydrogen or formate production with the above reduced electron carriers in a mechanism proposed for hydrogen generation in *Thermotoga maritima* [48]. In this mechanism the energetically favorable production of hydrogen or formate with reduced ferredoxin presumably provides the energetic input to enable the energetically unfavorable formation of hydrogen from NADH.

Strain MPOB^T^ ferments 7 fumarate to 6 succinate and 4 CO_2_ in pure culture, using the acetyl-CoA cleavage pathway to oxidize fumarate to CO_2_ [22]. All genes encoding for the acetyl-CoA cleavage pathway are present in the genome. In this pathway, acetyl-CoA is cleaved into a methyl-group and CO, which are both oxidized further to CO_2_. During propionate conversion, the pathway may be used anaplerotically to form acetyl-CoA. The genes for the acetyl-CoA pathway are scattered through the genome.

Acetyl-CoA is converted by an acetylCoA synthase/COdh complex encoded by Sfum_2564 – 2567 to CO_2_ and a methyl-group. The methyl group is further oxidized via 5,10-methylene tetrahydrofolate reductase (Sfum_3130), methylene-tetrahydrofolate dehydrogenase (S_fum 2686), methenyl-tetrahydrofolate cyclohydrolase (Sfum_1186), formyl-tetrafolate syntethase (Sfum_2687) and formate dehydrogenase to CO_2_. During complete oxidation of 1 fumarate to 4 CO_2_, 6 reducing equivalents are released that are used to reduce 6 fumarate to 6 succinate.

Strain MPOB^T^ is able to couple propionate oxidation to sulfate reduction [22]. The flow of electrons during respiratory sulfate reduction has not yet been fully described. As predicted, the genome encodes the full suite of genes necessary for dissimilatory sulfate reduction as well as several membrane complexes, which could deliver electrons from membrane electron carriers like menaquinol to cytosolic sulfate-reducing enzymes. Sulfate is first activated to adenosine-5′-phosphosulfate (APS) through the ATP dependent action of adenylylsulfate kinase. Two genes were detected that are predicted to code for adenylylsulfate kinase (Sfum_0774; 2338). Genes for APS reductase (Sfum_1047-48) were detected which likely encode the metabolic machinery necessary for the reduction of APS to sulfite and/or bisulfite. Sulfite and/or bisulfite are then reduced to sulfide by dissimilatory sulfite reductase. Alpha and beta subunits of the dissimilatory sulfite reductase were detected (Sfum_4042-43) as part of a predicted five-gene cluster along with genes that have high identity to dissimilatory sulfite reductase C (Sfum_4045). The gene organization of the alpha, beta and c-subunits of the whole cluster is different from other dissimilatory sulfate reducers [49]. Genes predicted to encode a membrane-bound dissimilatory sulfite reductase, *dsrMKJOP*, were detected (Sfum_1146-50) as were the three genes encoding a quinone-interacting membrane-bound oxidoreductase (Qmo) complex (Sfum_1285-87). Additionally, *qmoAB* genes were detected elsewhere on the chromosome as part of a larger multi-gene cluster (Sfum_1054-59) that contains genes predicted to code for a benzoyl-CoA reductase subunit A (Sfum_1051) and three additional hypothetical proteins of unknown function (Sfum_1052-54).

Other membrane complexes include a membrane-bound, ion-translocating ferredoxin:NADH oxidoreductase (Sfum_2694-99 gene product), which could drive the unfavorable formation of reduced ferredoxin from NADH by using the ion gradient, two NADH dehydrogenases (Sfum_0199-209 and Sfum_1935-43), and two pyrophosphatases (Sfum_2995 and Sfum_3037).

### Regulation and signal transduction

The *S. fumaroxidans* genome contains genes with similarity to those coding for a prototypical bacterial RNA core polymerase (RpoA, RpoB, RpoC) along with 12 sigma factors to confer promoter specificity. These sigma factors include one general housekeeping sigma 70 factor (RpoD), seven sigma 24 type stress related factors, two additional sigma 70-like factors, one FliA/WhiG sigma 28 type factor, and one 54 factor (RpoN) similar to that used for general nitrogen control in *Escherichia coli*. The genome also contains 31 genes with similarity to those coding for sigma 54-interacting transcriptional regulators (18 with response regulator signaling domains and 7 with PAS signaling domains, and 3 with GAF signaling domains), suggesting a major role for the 54-factor in global control of *S. fumaroxidans* gene expression. Numerous two-component regulatory systems (27 histidine kinase-type sensor transmitters, 11 response regulatory proteins, and 25 receiver-only domain proteins) are present in the genome. Compared to other Gram-negative microbes, *S. fumaroxidans* has a moderate number of primary transcription factors containing a helix-turn-helix motif (~115 genes).

### Motility and taxis

Unlike the thermophilic, syntrophic, propionate-utilizing, *Pelotomaculum thermopropionicum* SI [5] from the *Firmicutes*, *S. fumaroxidans* lacks genes coding for flagellar structural proteins (i.e., basal body, motor, hook, and filament) along with the associated flagellar biogenesis, anti-28 factor (FlgM) and the *E. coli* type master switch proteins, FlhCD or CheR, CheV, and CheC proteins. However, *S. fumaroxidans* contains genes for a FliA/WhiG family RNA polymerase sigma factor, nine methyl-accepting chemotaxis proteins (MCP) (three soluble and six membrane associated) with unknown roles in signal transduction plus one gene coding for each of the following signal processing proteins, CheA, CheW, CheY and CheD, and two *cheB* genes. Interestingly, genes for one PilQ type 4 secretion-like protein, two PilT-like retraction proteins, and PilM, PilY and PilO-like assembly proteins (one each) are predicted that may suggest alternative means of cell movement. Lastly, the absence of genes coding for pili-type nanowire proteins would suggest that direct interspecies electron transfer is unlikely [50].

### Host defense systems

To explore possibilities of developing a genetic system for *S. fumaroxidans* it is crucial to investigate the mechanisms that are present in the strain that protect against foreign DNA. The genome of strain MPOB^T^ contains two possible restriction-modification gene clusters. The first cluster consists of three genes, of which two encode the methylase and endonuclease of a Type-II restriction-modification system (Sfum_2532 and 2533, respectively) and shows high sequence identity with *PstI* and *BsuBI* restriction-modification systems. The third gene (Sfum_2534) encodes a modification requiring endonuclease which shows high sequence identity with the *E. coli* *mrr* gene, a modification-requiring restriction enzyme. The second cluster contains, two genes encoding a putative Type-III restriction modification system (Sfum_2855 – 2856) and three genes which do not seem to be part of the restriction-modification system. Interestingly, this system shows similarity only with hypothetical restriction-modification system coding sequences (nt-nt BLAST of both genes (endonuclease Sfum_2855 and methylase Sfum_2856): *Candidatus* Desulforudis audaxviator MP104C, 72% coverage; *Chlorobium limicola* DSM 245, 72% coverage; *Cellvibrio japonicus* Ueda107, 43% coverage; *Verminephrobacter eiseniae* EF01-2, 67% coverage). Since the genes do not show high sequence identity with described systems, it is not possible to predict the specificity of the system.

Additionally, the genome of strain MPOB^T^ harbors genes encoding two CRISPR/Cas systems. The first system (Sfum_1345-1356) can be classified as a type I-E system associated with a 69-spacer CRISPR locus, the second (Sfum_2824-2831) as type III-A system and is associated with a CRISPR locus containing 79 spacers [51].

Taken together, strain MPOB^T^ has multiple systems to protect itself against foreign DNA, making it a challenge to develop a genetic system for this strain.
